# Perirhinal Cortex Lesions in Rats: Novelty Detection and Sensitivity to Interference

**DOI:** 10.1037/bne0000049

**Published:** 2015-06

**Authors:** Mathieu M. Albasser, Cristian M. Olarte-Sánchez, Eman Amin, Malcolm W. Brown, Lisa Kinnavane, John P. Aggleton

**Affiliations:** 1 School of Psychology, Cardiff University; 2 MRC Centre for Synaptic Plasticity, Department of Physiology and Pharmacology, University of Bristol Medical School; 3 School of Psychology, Cardiff University

**Keywords:** habituation, hippocampus, learning, perirhinal cortex, recognition memory

## Abstract

Rats with perirhinal cortex lesions received multiple object recognition trials within a continuous session to examine whether they show false memories. Experiment 1 focused on exploration patterns during the first object recognition test postsurgery, in which each trial contained 1 novel and 1 familiar object. The perirhinal cortex lesions reduced time spent exploring novel objects, but did not affect overall time spent exploring the test objects (novel plus familiar). Replications with subsequent cohorts of rats (Experiments 2, 3, 4.1) repeated this pattern of results. When all recognition memory data were combined (Experiments 1–4), giving totals of 44 perirhinal lesion rats and 40 surgical sham controls, the perirhinal cortex lesions caused a marginal reduction in total exploration time. That decrease in time with novel objects was often compensated by increased exploration of familiar objects. Experiment 4 also assessed the impact of proactive interference on recognition memory. Evidence emerged that prior object experience could additionally impair recognition performance in rats with perirhinal cortex lesions. Experiment 5 examined exploration levels when rats were just given pairs of novel objects to explore. Despite their perirhinal cortex lesions, exploration levels were comparable with those of control rats. While the results of Experiment 4 support the notion that perirhinal lesions can increase sensitivity to proactive interference, the overall findings question whether rats lacking a perirhinal cortex typically behave as if novel objects are familiar, that is, show false recognition. Rather, the rats retain a signal of novelty but struggle to discriminate the identity of that signal.

Recognition memory is the ability to distinguish novel from familiar stimuli. Despite broad agreement that the perirhinal cortex is vital for object recognition memory in both rodents and primates (Brown & Aggleton, 2001; Mumby & Pinel, 1994; Murray, 1996; Murray, Bussey, & Saksida, 2007; Warburton & Brown, 2010; Winters, Saksida, & Bussey, 2008), there is debate over the nature of this involvement. One current model assumes that the perirhinal cortex holds object-level representations of complex stimuli that help to distinguish between stimuli with overlapping features (Bussey, Saksida, & Murray, 2005; Cowell, Bussey, & Saksida, 2010; Murray et al., 2007). Consequently, perirhinal cortex lesions force the use of simpler feature-based representations when discriminating stimuli. Thereby, perirhinal cortex lesions increase sensitivity to interference from related stimuli (Bartko, Winters, Cowell, Saksida, & Bussey, 2007b; Norman & Eacott, 2004), as the stimuli share more common features. This sensitivity causes novel objects to appear familiar, so disrupting recognition memory (Bartko, Winters, Cowell, Saksida, & Bussey, 2007a; Cowell et al., 2010; McTighe, Cowell, Winters, Bussey, & Saksida, 2010).

Striking evidence in support of this representation account comes from the finding that placing rats with perirhinal cortex lesions in a dark environment between object sample and recognition test, that is, during the retention period, can restore performance to that of control rats due to the release from proactive interference (McTighe et al., 2010). In that study, object recognition was assessed by comparing the levels of exploration given to two identical objects, when both objects were either novel or familiar. Normal rats showed heightened exploration of pairs of novel objects when compared with the pairs of familiar objects. In contrast, perirhinal cortex lesions caused reduced exploration of the novel objects, resulting in equivalent levels of exploration to both novel and familiar object pairs (McTighe et al., 2010). The latter finding was interpreted as showing that perirhinal lesions cause novel objects to be perceived as familiar, that is, produce false recognition (McTighe et al., 2010). Similar results have also been found for mice (Romberg et al., 2012).

Despite these findings, rats with perirhinal cortex lesions often show normal levels of exploration for novel objects on the sample trial used at the beginning of a spontaneous recognition test (Aggleton, Keen, Warburton, & Bussey, 1997; Albasser, Davies, Futter, & Aggleton, 2009; Albasser et al., 2011b; Barker, Bird, Alexander, & Warburton, 2007; Barker & Warburton 2011; Bartko et al., 2007a, 2007b; Ennaceur, Neave, & Aggleton, 1996; McTighe et al., 2010; Moran & Dalrymple-Alford, 2003; Mumby, Piterkin, Lecluse, & Lehmann, 2007; Winters, Forwood, Cowell, Saksida, & Bussey, 2004). For this pattern to occur, it has been argued that home cage conditions prior to testing constitute a low interference environment, that is, akin to being in the dark, so protecting performance despite the perirhinal cortex lesions (Romberg et al., 2012). It is also assumed that the objects encountered on the first sample trial can then be sufficient to produce marked interference effects on subsequent trials, so causing recognition deficits in these same rodents with perirhinal lesions (Romberg et al., 2012).

The present study examined these accounts by studying the exploration levels of rats with perirhinal lesions when confronted with a series of novel and familiar objects. The experiments all used the bow-tie maze (Albasser et al., 2010). This apparatus makes it possible to quantify changes in exploration levels across multiple trials within a single session, during which interference effects should accrue. The experimental data came from five separate studies (Cohorts 1–5), each containing rats with either perirhinal cortex lesions or sham surgeries. In Cohorts 1–4, the first postsurgery test of object recognition memory was conducted in the same standard way (Experiments 1–4). As this recognition test contained either 20 trials (Cohorts 1, 3, 4) or 10 trials (Cohort 2), it was also possible to combine the behavioral data to look for changes in overall levels of object exploration across many trials and many lesion cases. The initial session was selected as it would not suffer interference from previous recognition tests. Proactive interference should be least for the very first test trial but then accumulate at trials progress within a session, so increasingly disrupting performance by rats with perirhinal cortex lesions.

The rats in Cohort 4 were also studied when interference levels were manipulated more formally in order to assess their differential impact on rats with perirhinal cortex lesions (Experiments 4.2 and 4.3). Finally, Experiment 5 (Cohort 5) examined levels of exploration when rats only had pairs of novel objects to explore. In summary, the present study examined: (a) whether perirhinal cortex lesions reduce exploration levels consistent with treating novel objects as familiar, that is, suffering from false recognition, and, (b) whether perirhinal cortex lesions heighten sensitivity to interference.

## General Method

### Animals

All rats in the present study were male Lister Hooded rats (*Rattus norvegicus*) supplied by Harlan Olac (Bicester, United Kingdom). The rats were housed in pairs, with water provided ad libitum throughout the study. Animal husbandry and experimental procedures were conducted in accordance with the “Principles of Laboratory Animal Care” (NIH publication No. 85–23, revised 1985) and with the U.K. Animals (Scientific Procedures) Act, 1986, and associated guidelines, as well as EU directive 2010/63/EU. None of the animals were trained prior to surgery.

### Apparatus

**Figure 1 fig1:**
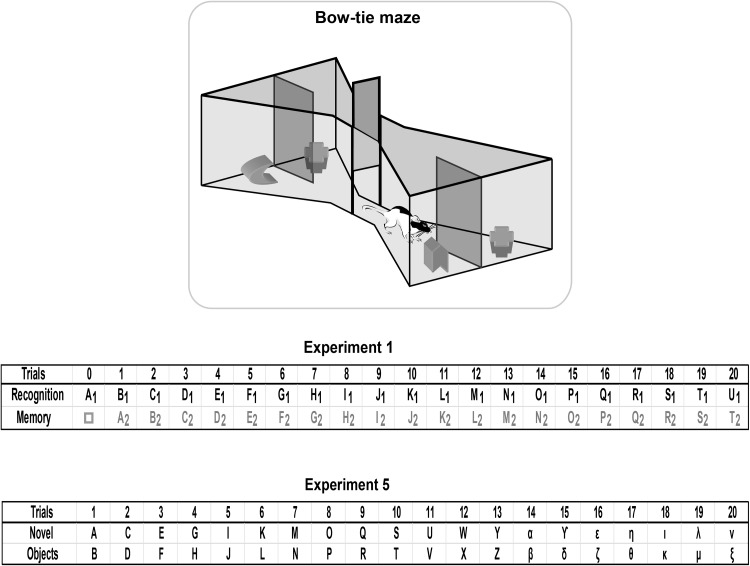
Schematic of the bow-tie maze (upper). A central sliding door separates the two ends of the maze in which objects are placed. The rat runs back and forth from end to end of the maze, with a new trial at each end (adapted from Albasser et al., 2011a). The sequence of object pairs in Experiment 1 (upper; object recognition) and Experiment 5 (novel object exploration is depicted). Different objects are represented by different letters while the subscript numbers in Experiment 1 show how duplicate objects were used for the recognition trial. For the first presentation of an object, that is, when novel, the letter is in bold. The □ symbol depicts the familiar object in Trial 0 that had also been used in pretraining. The left/right placement of novel objects was counterbalanced in Experiment 1. In Experiment 5, all objects were novel.

All experiments were conducted in a bow-tie shaped maze made with steel walls and a wooden floor (Figure 1 upper)—the “bow-tie maze.” The maze was 120 cm long, 50 cm wide, and 50 cm high. Each end of the apparatus was triangular, the apices of which were joined by a narrow corridor (12 cm wide). An opaque sliding door set in the middle of the corridor could be raised by the experimenter. The floor adjacent to the far wall of each triangle contained two recessed food wells, 3.5 cm in diameter and 2 cm deep. The food wells were separated by a short, opaque dividing wall that protruded 15 cm from the middle of the end wall. These food wells were covered by objects in the experiment proper. Illumination was provided by ceiling lights, giving a mean light intensity of around 580 lux in the center of the maze.[Fig-anchor fig1]

All experiments used numerous junk objects, each differing in shape, texture, size, and color. Every object was large enough to cover a food well but light enough to be displaced. Any object with an obvious scent was excluded. Sufficient objects were used to ensure that no object was repeated across experiments for the same cohort. All objects had multiple, identical copies, so that different copies of the same object were always used when an object was repeated within a session, so precluding scent marking. All objects were cleaned with alcohol wipes after each session.

### Pretraining

Prior to behavioral testing, the rats were reduced to 85% of their free feeding weight and maintained at this level throughout the experiment. On Day 1 of pretraining, pairs of rats were initially placed in the apparatus for 20 min where they explored the maze freely and ate sucrose pellets scattered on the floor and in the food wells (45 mg, Noyes Rodent Diet, Lancaster, NH). On Day 2, animals were placed in the maze singly for 10 min where only the food wells were baited. From Day 3, a single sucrose pellet was placed in each well, and individual rats were rewarded for shuttling between the two goal areas, that is, the wells were constantly rebaited for 10 min. The central sliding door controlled movement from one side of the maze to the other. Rats were then trained to displace two identical objects (initially wooden blocks) that covered the wells, so reaching the food rewards. Initially the blocks covered only one third of the well, so the reward was still visible. The blocks then moved progressively above the wells to eventually cover them. Pretraining was complete (Day 7) when rats would shuttle between the two ends of the maze as soon as the central door was raised and consistently displace an object over the well to reach a food reward. Three pairs of objects were used during pretraining, with two identical objects being placed at the same end of the maze on each pretraining trial.

## Experiment 1: Object Recognition (Short Delays)

### Materials and Method

Cohort 1 contained 18 rats housed under diurnal conditions (14 hr light/10 hr dark). At the time of surgery, these rats weighed between 290 g and 360 g. Recognition memory pretraining began 2 weeks after completion of a water maze visual discrimination task that consisted of three concurrent discriminations involving 6 two-dimensional stimuli (Aggleton, Albasser, Aggleton, Poirier, & Pearce, 2010).

#### Surgery

Both the perirhinal cortex lesion and sham surgical control rats were first injected with the analgesic Meloxicam (1.0 mg/kg) and then anesthetized with an intraperitoneal injection of sodium pentobarbital (60 mg/kg), before being placed in the stereotaxic frame (David Kopf Instruments, Tujunga, CA). The incisor bar was set at +5.0 mm to the horizontal plane. A sagittal incision was then made in the scalp, and the skin retracted to expose the skull. A dorsal craniotomy was made directly above the target region, and the dura cut to expose the cortex. The perirhinal lesions were made by injecting a solution of 0.09 M *N*-methyl-D-aspartic acid (NMDA; Sigma, Poole, United Kingdom) dissolved in phosphate-buffered saline (pH 7.4) using a 26 gauge, 1-μl Hamilton syringe (outside diameter 0.47 mm; Bonaduz, Switzerland). The stereotaxic coordinates of the three perirhinal lesion injections relative to ear-bar zero were (in mm): (a) antero-posterior (AP) 3.9, medial-lateral (ML) ± 5.9; (b) AP 2.4, ML ± 6.2; and (c) AP 0.7, ML ± 6.3. The dorso-ventral depth (DV), from bregma at the three sites was −9.3 (most rostral), −9.6, and −9.0 (most caudal). Injections of 0.20 μl of NMDA were made in the most rostral location, while the other two levels each received 0.19 μl NMDA. These injections were made at a rate of 0.10 μl per min with the needle left in place for a further 4 min after each injection. After every surgery, the skin was sutured together over the skull, and antibiotic powder was applied to the wound (Acramide, Dales Pharmaceuticals, United Kingdom). All animals received 5 ml of glucose saline subcutaneously, then placed in a heated box until they showed signs of recovery. Paracetamol (for pain relief) was dissolved in the rats’ drinking water for several days postsurgery. All surgeries were performed under aseptic conditions.

Ten rats received perirhinal cortex lesions (PRh1). Eight rats formed a surgical control group (Sham1). In all sham cases, the initial surgical procedure was as described above. After removal of a bone flap, the Hamilton syringe needle was lowered in 4 cases into the perirhinal region (coordinates as above), but without the injection of NMDA. In 4 other rats, the Hamilton syringe needle was inserted into the parietal cortex as a control for hippocampal surgeries (also examined in that study, see Aggleton et al., 2010).

#### Behavioral methods

Following pretraining (see General Method), the rats received one session of a standard spontaneous object recognition protocol that contained multiple test trials. During each test trial, the animal could freely explore two objects, one novel the other familiar, for a total of 1 min (see Figure 1). To start each session, a rat was placed on one side of the maze (Trial 0), where a single, novel object (object A_1_) covered a food well that contained a single sucrose pellet (45 mg; Noyes Purified Rodent Diet, Lancaster, NH). The other food well at that end of the maze also contained a single food reward, but this well was covered by a familiar object from pretraining (e.g., a wooden block, □). The rat remained in that part of the maze (with object A_1_) for 1 min. The central sliding door was then raised, and the rat ran to the opposite side of the maze to initiate Trial 1 (see Figure 1).

During Trial 1, the rat had a free choice between object A_2_ (the now familiar object) and novel object B_1_ (see Figure 1). Each object was concurrently available for the rat to explore for a total of 1 min. The central sliding door was then raised to reveal two more objects (the now familiar object B_2_ vs. novel object C_1_) at the opposite end of the maze for exploration by the rat (Trial 2). After a further 1-min period, the door was raised to reveal objects C_2_ and D_1_ (Trial 3), and so on to complete 20 trials. Thus, the retention period between trials was always a maximum of 60 s, but in reality it was typically much shorter. Both the familiar and the novel objects always covered a single 45 mg sucrose pellet, which were pushed aside to retrieve the pellet. This baiting procedure, which ensured that the objects were approached, did not affect the validity of the recognition test as this relied on the differential exploration of the novel and familiar objects (both of which were baited). The placement of objects (including novel objects) varied from left to right according to a pseudorandom schedule. The order of the particular objects used in the test was reversed for half of the rats. This counterbalancing ensured that the novel object in any given pair is alternated; for example, for half of the rats in the trial that paired together the following two objects, a toy and a can, the can was the novel object. For the remaining rats, the toy was the novel object. The set of objects used for each of the cohorts in this experiment varied.

#### Analysis of behavior

Animals were video-recorded throughout training. The scoring of each rat’s spontaneous exploration was blind, that is, the experimenter did not know group allocations until the experiment finished. Object exploration was defined as directing the nose at a distance <1 cm from the object, with the vibrissae moving, and/or touching it with the nose or the paws. Object exploration was not recorded when animals sat on the object, when rats used the object to rear upward with the nose of the rat facing the ceiling, or when chewing the object. The duration of exploration was determined by holding down a key pad on a computer during the bursts of exploration recorded on video. For tests of object recognition, two performance indices are often calculated, D1 and D2 (Ennaceur & Delacour, 1988). Index D1 is the duration of exploration time devoted to the novel object minus the exploration time devoted to the familiar object, that is, the extra time spent exploring the novel objects. The “cumulative D1” is the sum of the D1 scores across each trial. The second measure (D2) also uses the difference in exploration times (i.e., D1), but then divides D1 by the total duration of exploration given to both the novel and familiar objects, so adjusting for overall levels of exploration. This study focused on the D1 index as the key issues in the present study concerned total levels of exploration to novel and to familiar objects. The D1 index is readily derived from these measures (it is the difference), making it easy to see how these performance measures relate to each other.

To determine if either group successfully discriminated novel from familiar objects, we conducted one sample *t* tests based on the cumulative D1 recognition indices. These *t* tests assessed if the Group D1 scores were above chance (zero), that is, if the group showed a preference for novelty. Consequently, these were one-tailed tests. One-tailed tests also compared the recognition memory performance (D1) of the perirhinal cortex lesion group with that of the control group.

A further issue is that times spent with novel and familiar objects in the same trial are not independent as a rat cannot perform both behaviors simultaneously. For this reason, initial statistical analyses examined total exploration (novel plus familiar objects). This comparison was followed by separate analyses for novel and for familiar objects. All comparisons based on exploration times were two-tailed.

#### Histology

Following behavioral testing, all rats received a lethal overdose of sodium pentobarbitone (60 mg/kg, Euthatal, Rhone Merieux, United Kingdom) and were then transcardially perfused with 0.1 M phosphate buffer saline (PBS) followed by 4% paraformaldehyde in 0.1 M PBS (PFA). The brains were removed and postfixed in PFA for 4 hr and then transferred to 25% sucrose overnight at room temperature with rotation. Sections were cut at 40 μm on a freezing microtome or cryostat in the coronal plane and then stained with cresyl violet. Cytoarchitectural borders for the perirhinal cortex are from Burwell (2001).

**Figure 2 fig2:**
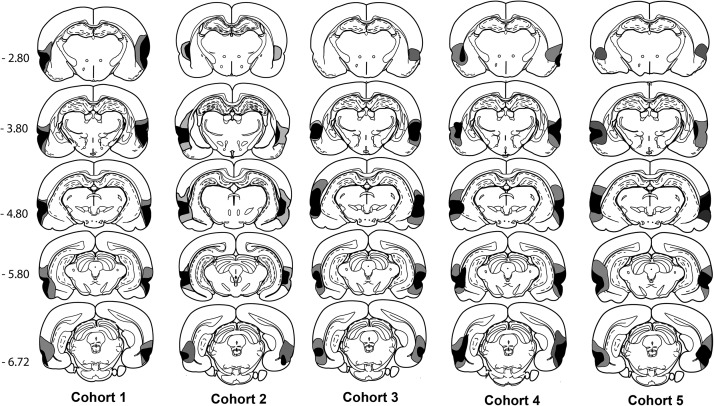
Diagrammatic reconstructions of the perirhinal cortex lesions showing the individual cases with the largest (gray) and smallest (black) lesions in each of the 5 experiments (Cohorts 1–5). The numbers refer to the distance (in mm) from bregma (from Figures 56, 65, 73, 81, 89 in Paxinos & Watson, 2005). Copyright 2005 by Elsevier Academic Press. Adapted with permission.

The lesions are represented on five equally spaced coronal sections that take in the length of the area (see Figure 2). Estimates of the percent damage to the perirhinal cortex in the cases with the largest and smallest lesions were calculated from these coronal reconstructions.[Fig-anchor fig2]

### Results

#### Histology

Of the 10 rats with perirhinal cortex lesions, one PRh1 rat became ill and was removed from the study. In the remaining animals (*n* = 9), the perirhinal cortex lesions were extensive, sometimes removing all of the target region (see Figure 2). Overall estimates of tissue loss in the perirhinal cortex ranged from 93% to 100%. One consequence was that in the largest lesions, the cell loss typically extended ventrally to involve dorsal and superficial parts of both the piriform cortex and lateral entorhinal cortex, often in both hemispheres. Most of the lesions involved the entire rostro-caudal extent of the perirhinal cortex, although in three cases there was limited, unilateral sparing of the perirhinal cortex at its most rostral border. In one case, there was some bilateral sparing in the upper part of area 36, that is, above the rhinal sulcus (see Figure 2). In seven cases, the perirhinal cortex lesion extended medially to cross the external capsule and cause a very restricted patch of cell loss in that part of caudal CA1 immediately adjacent to the fundus of the rhinal sulcus. In 6 cases, this localized CA1 damage was bilateral; in 1 other case, the cell loss was unilateral. In 2 cases, there was unilateral damage to the lateral nucleus of the amygdala at the rostral limit of the perirhinal cortex. In one of the sham control animals, there was appreciable, bilateral damage to the parietal cortex immediately above the dorsal hippocampus. This animal was removed from all analyses. These histological analyses left group numbers of PRh1 *n* = 9, Sham1 *n* = 7.

#### Object recognition and object exploration (20 trials)

**Figure 3 fig3:**
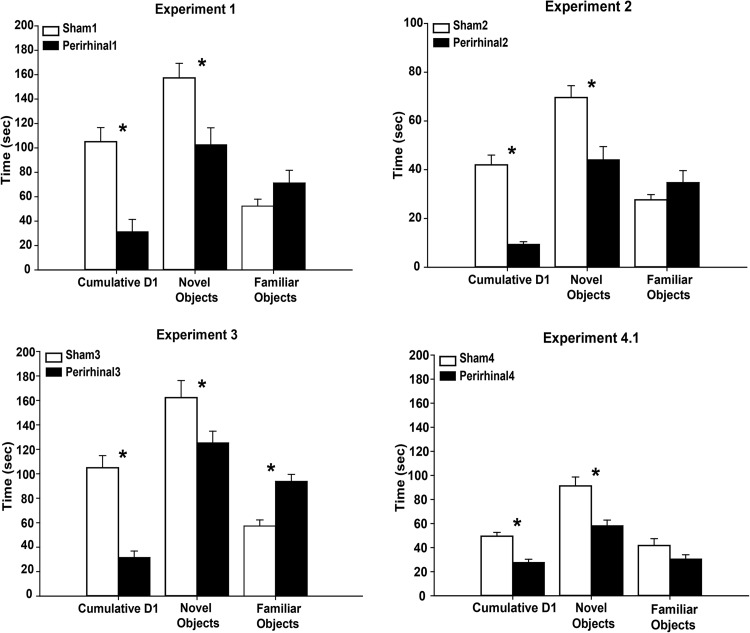
Recognition and exploration behavior of rats with perirhinal cortex lesions (PRh) and sham controls in 4 comparable experiments that gave rats multiple object recognition trials (each with one novel object and one familiar object) in a single session. The histograms (mean ± standard error) show the cumulative D1 recognition index (time with novel minus time with familiar) across all trials in the session. The separate mean total times spent with novel objects and familiar objects are also presented. Note, that Experiment 2 involved 10 trials, while Experiments 1, 3, and 4 involved 20 trials. * *p* < .05 (group difference).

Group comparisons based on the D1 recognition index (novel minus familiar object exploration times) accumulated over the entire session revealed a recognition memory deficit in the rats with perirhinal cortex lesions, *t*(14) = 4.79, *p* < .001 (Figure 3, top left). Both groups performed, however, above chance as demonstrated by one sample *t* tests; Sham1 (105.1 s) *t*(6) = 9.10, *p* < .001; PRh1 (31.2 s) *t*(8) = 3.07, *p* = .015.[Fig-anchor fig3]

Despite the difference in object recognition memory (D1), there was no group difference in the total exploration given to the novel and familiar objects; Sham1 *M* = 209.7 s, PRh1 *M* = 173.8 s, *t*(14) = 1.25, *p* = .23. Reflecting the D1 group difference, the Sham1 rats spent more time with novel objects (*M* = 157.4 s) than the PRh1 rats (*M* = 102.5 s), *t*(14) = 2.97, *p* = .012 (Figure 3, top left). Although more familiar object exploration was seen by the PRh1 group (*M* = 71.3 s) than the Sham1group (*M* = 52.3 s), this difference was not significant *t*(14) = 1.47, *p* = .16 (Figure 3, top left).

## Experiment 2: Object Recognition (Short Delays)

This study provided a replication of Experiment 1. The object recognition test was now the first postoperative study, pretraining starting a minimum of 2 weeks after surgery.

### Materials and Method

#### Housing and surgery

The housing conditions for Cohort 2, which comprised 26 rats, were identical to those described for Experiment 1. At the time of surgery, these rats weighed between 282 g and 322 g. Sixteen rats received bilateral perirhinal cortex lesions (PRh2) while 10 rats served as controls (Sham2). The surgical procedure was modified slightly from that in Experiment 1. The rats were anesthetized using an isoflurane-oxygen mixture before being placed in a stereotaxic frame. Injections of 0.22 μl NMDA were made in the following sites, relative to bregma (in mm); (a) AP −1.8, ML ± 5.9, DV −9.3; (b) AP −3.4, ML ± 6.1, DV −9.6, (c) AP −5.0, ML ± 6.2, DV −9.0. Rats in the surgical control group received identical treatment except that the dura was repeatedly perforated with a 25-gauge Microlance 3 needle (Becton Dickinson, Drogheda, Ireland), and no fluid was infused into the brain. Postoperative care corresponded to that in Experiment 1.

#### Behavioral methods

The testing procedures were identical to those described in Experiment 1 with the single exception that the rats in Cohort 2 only received 10 trials in their first test of object recognition after surgery. All other methods matched Experiment 1.

### Results

#### Histology

Of the 16 PRh2 rats, 4 were excluded as they had excessive cortical damage (*n* = 3) or only unilateral perirhinal cortex damage (*n* = 1). The remaining 12 PRh2 surgeries removed a considerable extent of perirhinal cortex (see Figure 2). Overall estimates of tissue loss in the perirhinal cortex ranged from 64% to 95%. Among these 12 cases, the only region with sparing in the majority of cases was the very rostral border of the perirhinal cortex, a region outside the perirhinal cortex as designated by Shi and Cassell (1999). In contrast, the mid and caudal perirhinal cortex showed extensive bilateral cell loss in all 12 cases. Reflecting the completeness of these lesions, there was additional involvement of the dorsal and superficial parts of the piriform and lateral entorhinal cortices, often in both hemispheres. Ventral area Te2 was sometimes thinned (*n* = 7). In 8 rats, the lesions extended medially to involve a very restricted portion of caudal CA1, immediately medial to the fundus of the rhinal sulcus (bilateral in 2 cases). The lesions occasionally extended unilaterally onto more superior cortex (*n* = 4). In 5 cases, there was unilateral damage to rostral postrhinal cortex. These histological analyses left group numbers of PRh2 *n* = 12, Sham2 *n* = 10.

#### Object recognition and object exploration (10 trials)

As in Experiment 1, the PRh2 rats had lower recognition memory (D1) scores than the Sham2 when summed over the entire session, *t*(20) = 8.51, *p* < .001 (Figure 3, top right). Both groups, however, performed above chance: Sham2 (mean D1 = 42.0 s) *t*(9) = 10.5, *p* < .001, PRh2 (mean D1 = 9.36 s), *t*(11) = 8.61, *p* < .001.

While the total time spent exploring objects (novel plus familiar) was higher in the Sham2 group, this difference was not significant: Sham2 (mean 97.3 s) PRh2 (mean 78.8 s), *t*(20) = 1.45, *p* = .16. The Sham2 rats spent more time exploring the novel objects (mean 69.6 s) than the PRh2 rats (mean 44.1 s), *t*(20) = 3.45, *p* = .003 (Figure 3, top right). While the PRh2 group spent more time overall exploring the familiar objects (*M* = 34.7 s) than the Sham2 rats (*M* = 27.6 s), this group difference was not significant *t*(20) = 1.23, *p* = .23 (Figure 3, top right).

## Experiment 3: Object Recognition (Short Delays)

This study provided a further replication of Experiments 1 and 2, with behavioral training identical to that described for Experiment 1.

### Materials and Method

Cohort 3, which comprised 24 rats, was maintained as described for Experiment 1, except that the rats were kept on a 12 hr:12 hr light to dark cycle. The weights of the rats at the time of surgery ranged from 273 g to 343 g. Twelve rats received bilateral perirhinal cortex lesions (PRh3) while 12 rats served as controls (Sham3). All rats in Cohort 3 were first anesthetized using an isoflurane-oxygen mix. Injections of 0.25 μl NMDA were made through a 2-μl Hamilton syringe held with a microinjector (Kopf Instruments, Model 5000). The procedure and coordinates of the injection sites were the same as Cohort 2, as were the surgical procedures for the control group. Behavioral training for recognition memory followed completion of a water maze task that involved discriminating plain walls of different lengths (Horne et al., 2010). The object recognition test was run exactly the same as Experiment 1 (i.e., 20 trials).

### Results

#### Histology

Two rats with perirhinal lesions were excluded from the behavioral analysis due to additional cell loss that included the auditory and visual cortices in both hemispheres, parts of the CA1 field, as well as excessive ventral damage including much of the piriform cortex. The largest and smallest of the perirhinal lesions (PRh3) in the remaining animals are depicted in Figure 2. The overall estimates of perirhinal tissue loss in these cases was 90% and 74%, respectively. The perirhinal lesions were centered on the rhinal sulcus and extended from the mid level of the amygdala to the posterior hippocampus. All rats sustained very extensive damage to the perirhinal cortex (areas 35 and 36) as well as partial damage to the lateral entorhinal cortices and the temporal association cortex immediately above area 36 (area Te2). Eight rats sustained some damage to the deep layers of the auditory association cortices. The dorsal piriform cortex was affected in all rats. All rats had sparing of the external capsule, the subiculum, and rest of the hippocampus. The numbers of rats in each group after histological assessment were PRh3 *n* = 10, Sham3 *n* = 12.

#### Object recognition and object exploration (20 trials)

The object recognition index (D1) scores were significantly lower in the PRh3 rats than in their respective sham controls, *t*(20) = 6.14, *p* < .001 (Figure 3, bottom left). The D1 scores of both groups were, however, above chance: Sham3 (*M* = 104.9 s), one sample *t* test, *t*(11) = 10.6, *p* < .001, PRh3 (*M* = 31.5 s), *t*(9) = 5.92, *p* < .001.

The total time spent exploring objects across the 20 trials did not differ between the two groups (*t* < 1); Sham3 *M* = 219.6 s, PRh3 *M* = 219.1 s. The Sham3 rats spent more time exploring novel objects (*M* = 163.2 s) than the PRh3 rats (*M* = 125.3 s), *t*(20) = 2.09, *p* = .05 (Figure 3, bottom left). In contrast, familiar object exploration was significantly higher by the PRh3 rats (*M* = 93.8 s) than the Sham3 rats (*M* = 57.3 s), *t*(20) = 4.83, *p* < .001 (Figure 3, bottom left).

## Experiment 4: Changing Levels of Visual Interference on Object Recognition

This experiment began with a further replication of Experiments 1–3, in order to increase the power of the combined analyses (Experiment 4.1). In addition, two further experiments examined whether rats with perirhinal cortex lesions are especially sensitive to proactive interference.

### Materials and Method

Cohort 4 consisted of 28 rats with a weight range of 295 g to 316 g at the time of surgery. The housing conditions matched those of Cohort 3. The surgical procedures for Cohort 4 were identical to those described for Cohort 2.

### Experiment 4.1 Standard Object Recognition

#### Behavioral procedure

The experiment involved the same standard object recognition task used in Experiments 1–3, with the rats receiving 20 trials. The object recognition test was the first postoperative study, pretraining starting a minimum of 2 weeks after surgery.

#### Combined statistical analysis (Cohorts 1–4)

In addition to reporting the behavioral findings for Cohort 4, it was possible to combine data from across all 4 experiments in order to examine, with an unusually large total number of animals, some key questions. Statistical comparisons involving exploration and discrimination behavior by the four cohorts, each of which contained two surgical groups (perirhinal or sham), used a mixed analysis of variance with between factors (surgery and cohort) and one within factor (early or late trial position).

### Experiment 4.2 Recognition Memory After the Presence or Absence of an Intervening Object

#### Behavioral procedure

Following Experiment 4.1, Cohort 4 received additional recognition tests in the bow-tie maze. The first set of tests involved switching from the light to the dark between trials (not reported here). Two further experiments (4.2 and 4.3) examined the consequences of varying visual interference on recognition memory performance. Experiment 4.2 began 4 weeks after Experiment 4.1.

**Figure 4 fig4:**
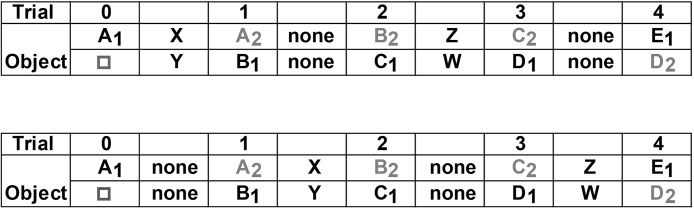
Schematic diagram showing the two trial types in Experiment 4.2, which varied interference levels. For one half of the trials, the rats had just explored a preceding pair of novel objects (intervening objects, e.g., Trial 1, upper), while for the remaining trials, there was no immediately preceding object (none, e.g., Trial 2, upper). Each letter corresponds to an object, with novel objects in bold. The object numbers show how duplicate (or triplicate) objects were used for the recognition trials. The □ symbol depicts the familiar pretraining object used for Trial 0. The columns signify the alternating ends of the bow-tie maze, so that on Trials 0, 1, 2, 3, and 4, the rat returns to the same end of the maze. All rats received both session types, with different objects used in the two sessions.

To begin a session in Experiment 4.2, the rat was placed at one end of the bow-tie maze, which contained a novel object (A_1_) and a familiar wooden block (□) from pretraining, each placed over a food well (Trial 0, Figure 4). After 1 min, the central door was raised, and the rat ran to the other end of the maze to find two more food pellets. On alternating trials, the food wells were covered with either two novel, but different, objects (*X, Y*), or by no objects at all (none, Figure 4). After 1 further min, the central door was raised so that the rat returned to the original (Trial 0) end of the maze to find more food pellets (Trial 1). One food well was covered by a copy of the now familiar object (A_2_), while a novel object (B_1_) covered the other well, so providing an object recognition trial. This sequence was repeated for multiple trials, half of the trials with intervening objects in the 1-min retention period, and the other half with no intervening objects (none, see Figure 4).[Fig-anchor fig4]

A counterbalanced version of the test was given 14 days later, so that those rats which had explored interfering objects (*X, Y*) between Trial 0 and Trial 1 now received a corresponding first trial with no intervening objects (none; see Figure 4). In this way, the effects of interference between sample and test were compared. Although rats received multiple trials, only the first 4 trials are considered in the results as: (a) any differences between the two conditions (intervening objects vs. none) should diminish over successive trials with the increase in interference, and (b) the focus on the initial trials provides a more direct comparison with Experiment 4.3, which only involved 4 trials.

### Experiment 4.3 Pretest Manipulations of Interference Levels (Waiting in the Light or in the Dark)

#### Behavioral procedure

**Figure 5 fig5:**
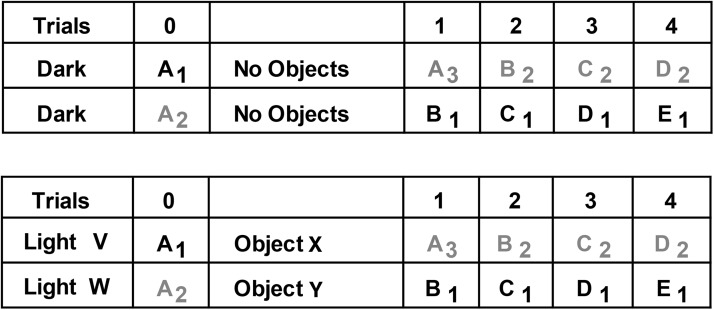
Schematic of the low interference (upper) and high interference (lower) conditions used in Experiment 4.3. For the low interference session, the rat was kept in the dark prior to testing. For the high interference session, the rat had been kept in a cage in the light that contained two novel objects (V, W). These conditions were followed by a familiarization trial with two copies of novel object A (Trial 0). For the high interference condition, two objects (X,Y) were presented between Trial 0 and Trial 1. The remaining trials matched the standard object recognition protocol. Each letter corresponds to an object, with novel objects in bold. The object numbers show how duplicate (or triplicate) objects were used for the recognition trials. The columns reflect the alternating ends of the bow-tie maze. All rats received both session types, with different objects used in the two sessions.

Each rat was trained on two conditions (*low interference* and *high interference*), which were counterbalanced for presentation order (see Figure 5). Each testing condition was separated by 2 days. Rats received 4 trials on each condition, with the expectation that the initial trials would show any differential effects of prior interference (see Figure 5).[Fig-anchor fig5]

For the low interference condition, each rat was taken individually from the holding room in the standard metal carrying box, which is light proof, to a waiting room that is different to the test room. (All rats had previously been habituated to the waiting room by being placed in that room for 1 hr on each of 2 days prior to the start of the experiment.) The rat was placed, in the dark, in an individual holding cage (the same dimensions as the home cage) and remained there for 1 hr. After 1 hr, the rat was placed in the carrying box (in the dark), taken to the test room (in the dark), and placed in the maze to start the experiment (Figure 5, upper).

For the high interference condition, each rat was taken individually from the holding room in a metal carrying box to the waiting room, where it was placed in a holding cage that the rats had previously experienced (see above). The waiting room was illuminated by room lights (580 lux in the room center). Two different novel objects (*V* and *W*; see Figure 5, lower) had been placed in the holding cage, so these could be explored by the rat. The rat remained in the holding cage with the objects for 1 hr. The rat was then placed back in the carrying box and taken to the test room (which was in the light) to start the experiment (Figure 5, lower).

The subsequent object recognition tests used the standard test procedure described above for Experiment 1, that is, 1 min at each end of the maze, with each trial consisting of one novel object and one familiar object (from the previous trial). The only differences were that: (a) identical copies of a novel object were used for Trial 0, and (b) a pair of novel objects (*X, Y*) was placed at the opposite end of the maze between Trials 0 and 1 for the high interference group to explore for up to 1 min (see Figure 5).

### Results

#### Histology

Three of the 16 perirhinal lesion cases were rejected, as 1 had bilateral hippocampal cell loss, 1 had sparing of the upper bank of the rhinal sulcus in one hemisphere, while a third suffered particularly extensive cortical damage beyond perirhinal cortex. In the remaining 13 cases, cell loss throughout the anterior-posterior extent of the perirhinal cortex (both areas 35 and 36) was very consistent (see Figure 2). Overall estimates of perirhinal tissue loss in the remaining 13 cases ranged from 82% to 100%. At their caudal limit, the lesions consistently reached the rostral part of the postrhinal cortex. In addition to the perirhinal cortex, there was consistent cell loss in the lateral entorhinal cortex (especially the superficial cell layers), which sometimes involved the caudal parts of the piriform cortex. Some limited, unilateral cell loss was seen in the lateral amygdala nucleus in most cases. The perirhinal lesions often reached into the ventral part of Te2 but never extended above this area. In these 13 cases, it was typical for the lesion to include a small region of CA1 cells adjacent to the fundus of the rhinal sulcus, but any hippocampal cell loss was either unilateral (*n* = 8) or extremely restricted when bilateral (*n* = 5). One sham rat was removed from analyses, as there was ischemic damage in the right hemisphere. The final group numbers were, therefore, PRh4 *n* = 13, Sham4 *n* = 11.

#### Experiment 4.1 standard object recognition and object exploration (20 trials)

Group comparisons using the D1 recognition index (novel minus familiar object exploration times) accumulated across the session revealed the poorer recognition performance by the PRh4 rats, *t*(22) = 5.25, *p* < .001 (Figure 3, bottom right). Both groups did, however, still prefer the novel to the familiar object: Sham4 (mean D1 = 49.5 s), one sample *t*(10) = 15.6, *p* < .001, PRh4 (mean D1 = 27.6 s), *t*(12) = 10.2, *p* < .001.

The Sham4 group spent more time in overall (novel plus familiar) exploration over the 20 trials than the rats with perirhinal cortex lesions: Sham4 (*M* = 133.0 s), PRh4 (*M* = 88.7 s), *t*(22) = 3.04, *p* = .006. The Sham4 group also spent more time exploring the novel objects: Sham4 (*M* = 91.2 s) PRh4 (*M* = 58.2 s), *t*(22) = 3.87, *p* < .001 (Figure 3, bottom right). Likewise, familiar object exploration was higher by Sham4 group (*M* = 41.8) than PRh4 group (*M* = 30.5 s), but this difference was not significant, *t*(22) = 1.74, *p* = .096 (Figure 3 bottom right).

#### Experiments 1–4 standard objects recognition—Combined analyses for Cohorts 1–4

Combined, the 4 experiments provided a total of 44 perirhinal cortex lesion cases and 40 surgical controls. Although there were some minor differences in the surgical procedures and whether the rats had been trained on other behavioral tasks, all 4 cohorts received identical object recognition testing. For this reason, the cohort data were analyzed jointly, with cohort retained as a factor. Cohort 2 only received 10 trials, while the remaining 3 cohorts received 20 object recognition trials during this first session.

The principal comparisons examined the times spent exploring the novel and familiar objects. Of particular interest was how these times might be affected by the lesions and by the progression of trials across a session, as proactive interference effects should increase. Although main effects are reported, simple effects are often omitted, as for some data there were interactions across cohorts. It should be noted that Trial 1 is slightly different to all of the following trials within the same session (see Figure 1). The principal difference is that the sample phase (Trial 0) immediately prior to the first trial involves a novel object paired with a highly familiar item from pretraining, for example, a wooden block. In all subsequent trials, the familiar object is the novel object from the preceding trial (see Figure 1). For this reason, most analyses excluded Trial 1. In view of the large number of potential analyses, only key comparisons are presented.

(1) Do perirhinal lesions affect object exploration performance when proactive interference is lowest in the study? (First trial, all 4 cohorts.)

##### Trial 1

**Figure 6 fig6:**
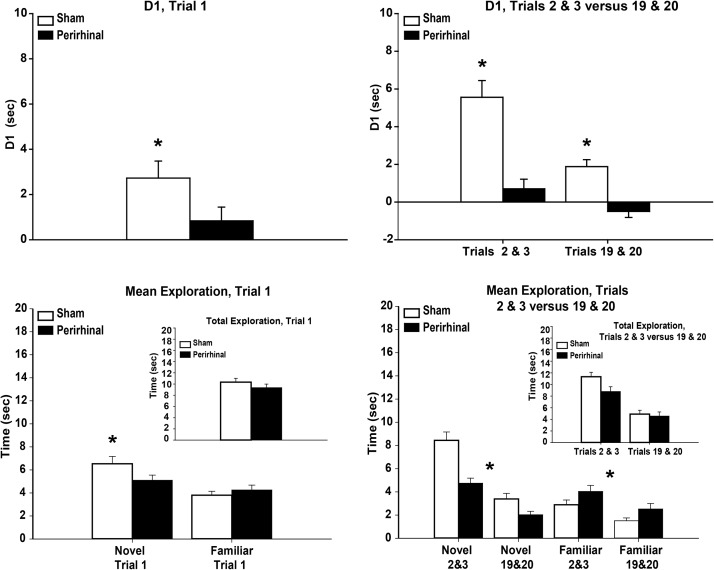
Experiments 1–4, combined data: Object recognition performance by combined cohorts of rats with perirhinal cortex lesions (black) and their sham controls (white). For all cohorts, one novel and one familiar object were presented on each of 10 or 20 consecutive trials. The histograms show the Trial 1 recognition discrimination ratio D1 (upper left; time spent with novel minus time spent with familiar). The mean exploration levels for the novel and familiar objects from Trial 1, with total object exploration (lower left). The Trial 1 data are from Cohorts 1–4. The D1 recognition memory scores for Trials 2 + 3 and Trials 19 + 20 (upper right). The mean exploration times for each pair of trials for the novel and familiar objects, with total object exploration (lower right). These data (Trials 2 + 3, 19 + 20) are combined from Cohorts 1, 3, and 4. Data shown are mean ± standard error. * *p* < .05 (those asterisks between histograms signal main effects of lesion).

The first trial of the first object recognition test postsurgery should suffer the least proactive interference when contrasted with later trials. The perirhinal cortex lesions (*n* = 44) impaired the D1 index of recognition memory when just this first trial is considered: *F*(1, 76) = 5.67, *p* = .020 (Figure 6, upper left). Unlike the control rats (*n* = 40), the combined D1 scores of the perirhinal lesion rats did not differ significantly from the chance score of zero, *t*(43) = 1.38, *p* = .087.[Fig-anchor fig6]

The combined rats with perirhinal lesions displayed comparable total levels of exploration to the control rats on Trial 1, *F*(1, 76) = 1.31, *p* = .26 (Figure 6, lower left). Separate analyses showed that the control rats spent more time exploring the novel object during Trial 1 than the rats with perirhinal lesions, *F*(1, 76) = 4.70, *p* = .033 (Figure 6, lower left). No group difference was found for time spent exploring the familiar object, *F*(1, 76) = 1.39, *p* = .24. In summary, the perirhinal lesions impaired recognition performance from the very first postoperative trial. While this lesion-induced deficit was accompanied by a decrease in exploration of the novel object, there was no overall difference in total object exploration.

(2) Do perirhinal lesions differentially affect object exploration across a test session as proactive interference increases? (Pairs of trials, 3 cohorts.)

This analysis considered data from adjacent pairs of trials from early and late stages of the same session. By combining adjacent trials, the impact of individual trial variability would be reduced, while still allowing examination of the most extreme placed trials. For reasons already given, Trial 1 was not included as it is procedurally different to all other trials.

##### Trials 2 + 3 versus Trials 19 + 20

Three cohorts received 20 trials in the first session. The perirhinal lesions reduced overall D1 scores for the combined Trials 2 + 3 and Trials 19 + 20, *F*(1, 56) = 48.61, *p* < .001 (Figure 6, upper right). There was also a clear decline in overall discrimination performance on Trials 19 + 20 when compared with the earlier Trials 2 + 3, *F*(1, 56) = 16.44, *p* < .001. The presence of a lesion by trial position interaction on the D1 scores, *F*(1, 56) = 5.17, *p* = .027, suggested a relatively greater drop in recognition performance by the control rats on the final two trials (Figure 6, upper right). This interaction is, however, compromised by the higher initial D1 scores of the control rats, making it difficult to interpret (Figure 6, upper right).

The total exploration times for these two pairs of trials (2 + 3, 19 + 20) did not differ between the two surgical groups, *F*(1, 56) = 2.43, *p* = .13 (Figure 6, lower right). There was, however, a relative decrease in overall exploration for the last two trials, *F*(1, 56) = 99.08, *p* < .001, which interacted with surgical group, *F*(1, 56) = 4.27, *p* = .044 (Figure 6, lower right). This interaction reflected the greater decline in overall exploration levels by the control rats for the final pair of trials. Simple effects also showed that the sham rats explored more overall on Trials 2 + 3.

Separate analyses for the novel objects showed higher overall exploration levels by the control groups, *F*(1, 56) = 19.5, *p* < .001, with an effect of trial position, *F*(1, 56) = 84.9, *p* < .001, as exploration levels declined by the end of the session. This decrease in exploration of novel objects interacted with lesion, *F*(1, 56) = 8.83, *p* = .004, with the sham rats showing the greater decrease (Figure 6, lower right). This interaction is, however, affected by scaling effects as the sham rats started at a higher level of exploration. In contrast, the perirhinal lesion rats displayed greater exploration of the familiar objects than the sham controls, *F*(1, 56) = 10.2, *p* = .002, with an overall decrease in exploration by both groups by the end of the session, *F*(1, 56) = 14.8, *p* < .001, with no interaction by lesion, *F* < 1.

In summary, both the perirhinal lesioned and sham control rats showed a decrease in exploration levels for the final trials, but this decrease was not enhanced by the perirhinal lesions. While the control rats showed greater exploration levels of novel objects, this difference was counterbalanced by the rats with perirhinal lesions showing a relative increase in the exploration of familiar items (Figure 6, lower right). This pattern extended to the final pair of trials (19 + 20) as there was no overall group difference in exploration (F < 1), but a relative decrease in novel object exploration *F*(1, 56) = 5.29, *p* = .025, countered by a relative increase in familiar object exploration, *F*(1, 56) = 4.35, *p* = .042, resulting in a D1 index difference, *F*(1, 56) = 23.6, *p* = .001.

(3) Do perirhinal lesions differentially affect object exploration across a test session as proactive interference increases? (Two blocks of 5 trials, all 4 cohorts.)

The purpose of blocking the data by sets of 5 adjacent trials was twofold. The first was to take advantage of all available data when comparing the impact of increasing trial position. The second was that for many of the previous comparisons the D1 scores from the rats with perirhinal lesions were not above chance. To help combat floor effects associated with recognition, trial scores were added across 5 consecutive trials (rather than 2 trials, as above). Grouped data comparisons showed that the cumulative D1 scores of the perirhinal rats were now above chance until the final block: Trials 1–5, *t*(43) = 5.34, *p* < .001; Trials 6–10, *t*(43) = 5.43, *p* < .01; Trials 11–15, *t*(31) = 5.20, *p* < .001; and Trials 16–20, *t* < 1.0.

##### Trials 1–5 versus Trials 6–10

The two blocks of 5 trials used the data from all 44 perirhinal lesioned rats and all 40 sham rats (data not presented graphically). The rats with perirhinal lesions performed poorly overall on the recognition measure D1, *F*(1, 76) = 85.6, *p* < .001, though there was no overall effect of trial position, D1, *F* < 1; D2, *F*(1, 76) = 2.68, *p* = .11, and no interaction with lesion, *F* < 1.

For total exploration times (novel plus familiar), there was no effect of trial position, *F* < 1, but there was a borderline effect of lesion, *F*(1, 76) = 4.02, *p* = .049, as the perirhinal lesion rats explored slightly less overall. There was no trial block by lesion interaction on this measure, *F*(1, 76) = 2.25, *p* = .14. Analyses based on just the novel or just the familiar objects showed the same pattern found in the previous section. That is, the sham rats spent more time exploring the novel objects than the perirhinal rats, *F*(1, 76) = 27.5, *p* < .001, while the perirhinal lesion rats spent more time exploring the familiar objects than the sham rats, *F*(1, 76) = 14.6, *p* < .001. There was no effect of trial block for either novel or familiar objects, both *F* < 1, and no interaction with surgical group; novel objects, *F*(1, 76) = 1.01; familiar objects, *F*(1, 76) = 2.94, *p* = .091.

In summary, the combined exploration data revealed a small overall decrease in object exploration by rats with perirhinal lesions, though they also showed greater exploration of the familiar objects than the sham rats. These behaviors did not appear to be affected by trial position.

(4) Do perirhinal lesions differentially affect object exploration across a test session as proactive interference increases? (Four blocks of 5 trials, 3 cohorts.)

##### Trials 1–5, 6–10, 11–15, 16–20

**Figure 7 fig7:**
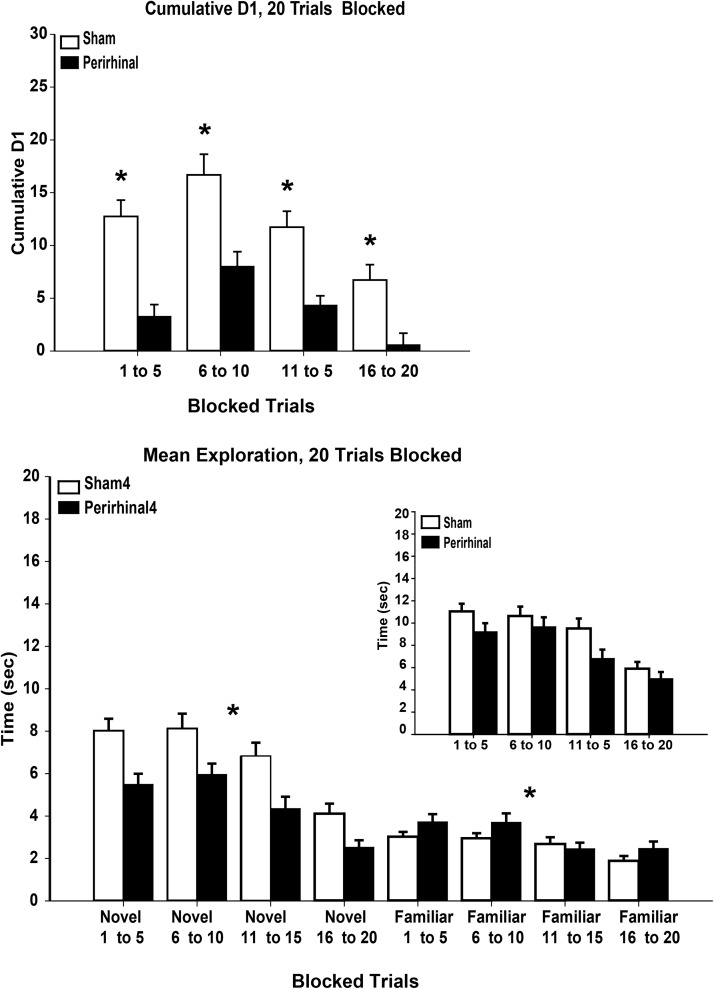
Experiments 1, 3, and 4, combined data from Trials 1–20, in four blocks of 5 trials. Object recognition (upper) and exploration (lower) by 3 cohorts of rats with perirhinal cortex lesions (black) and their controls (white). One novel and one familiar object was presented on each of 20 trials. The upper histograms depict recognition memory performance (D1) across four consecutive blocks of 5 trials. The lower histograms show the separate exploration levels for the novel and familiar objects in Trials 1–20, grouped into four blocks of 5 trials. The inset shows the combined times for novel plus familiar objects. Data shown are mean ± standard error of the mean. * *p* < .05 (those between histograms signal main effects of lesion).

When the data from all 20 trials were combined (see Figure 7), the pattern was similar to that seen in previous analyses. While the perirhinal lesions impaired recognition and reduced exploration of novel objects, these same rats often showed more exploration of the familiar objects than the sham control rats. The net result was a small overall decrease in exploration following perirhinal cortex lesions (Figure 7, lower). There was no evidence that the increase in proactive interference associated with the later trials had an exaggerated impact on the rats with perirhinal lesions.[Fig-anchor fig7]

The perirhinal lesions reduced the cumulative D1 scores, *F*(1, 56) = 85.5, *p* < .001 (Figure 7, upper). There was also a clear effect of trial position as recognition performance on later trials declined when compared with earlier blocks of trials, *F*(3, 168) = 24.8, *p* < .001. While there was no lesion by trial position interaction, *F*(3, 168) = 1.88, *p* = .14, this comparison is limited by scaling effects.

When total exploration times were compared across the four blocks of 5 trials, there was a decrease in overall exploration by the rats with perirhinal lesions when compared with the sham rats, *F*(1, 56) = 4.36, *p* = .041. (In none of the individual blocks of trials was this difference significant according to simple effects.) Across the 3 cohorts, there was a highly significant effect of trial block, with lower overall exploration levels (novel plus familiar) for the final block of trials, *F*(3, 168) = 37.6, *p* < .001 (Figure 7). There was no trial block by lesion group interaction for total exploration, *F*(3, 168) = 1.50, *p* = .22, as the decline in exploration by the last block of trials appeared equivalent for the two sets of animals (Figure 7).

Separate analyses for the novel objects showed that the control rats explored more than the perirhinal rats, *F*(1, 56) = 23.1, *p* < .001, and that there was an overall decrease in exploration by the final block of trials, *F*(3, 168) = 37.4, *p* < .001, but no interaction between these measures, *F* < 1 (see Figure 7). Parallel analyses for the familiar objects showed that the perirhinal rats explored more than the sham rats, *F*(1, 56) = 8.60, *p* = .005, with an overall decrease by the final block of trials, *F*(3, 168) = 12.8, *p* < .001 (Figure 7, lower). There was also a significant lesion by trial block interaction, *F*(3, 168) = 4.43, *p* = .005, reflecting how the perirhinal groups showed more familiar object exploration than the sham rats for the first two blocks of 5 trials, but this group difference in exploration levels disappeared for the final two blocks of trials.

The reduction in D1 scores with later trials raises the question of whether this effect was due to lower levels of overall exploration (see Figure 7), lower discrimination levels, or both. For this reason, the D2 index was compared across these four blocks of 5 trials. As with D1, the D2 scores were reduced in the animals with perirhinal lesions, *F*(1, 56) = 109.8, *p* < .001, while rats across both groups showed reduced D2 scores by the final block of trials (effect of block, *F*(3, 168) = 16.1, *p* < .001). There was, consequently, no interaction for D2 between trial block and lesion group, *F* < 1, with the profile of D2 scores across the four blocks appearing similar for the combined shams and for the combined perirhinal groups. At the same time, the D2 scores were consistently lower in the latter grouping.

#### Experiment 4.2 recognition memory after the presence or absence of an intervening object

**Figure 8 fig8:**
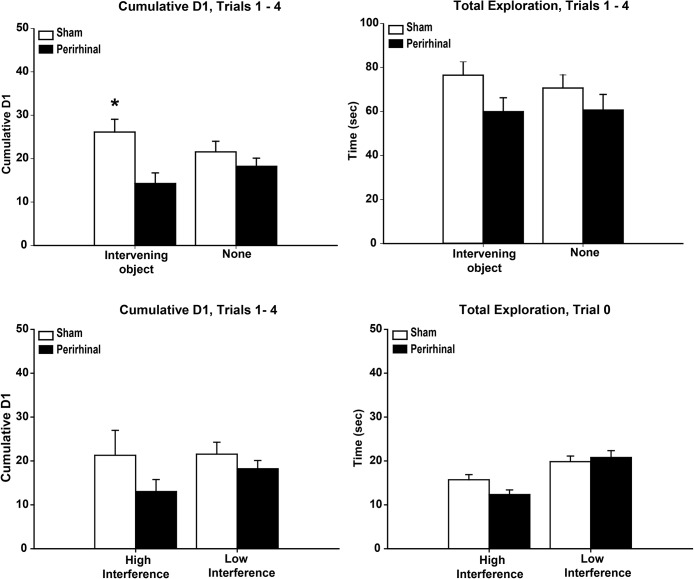
Experiments 4.2 and 4.3, perirhinal cortex lesions and interference (Cohort 4). Experiment 4.2 (upper). The histograms show the cumulative recognition index (D1) scores (Trials 1–4, upper left) and total exploration times (upper right) of rats with perirhinal lesions (black) and their controls (white) on two object recognition conditions that compared interference effects. In one condition, the rats explored novel objects between each recognition trial (intervening object); in the other condition, there were no intervening objects between trials (none). Experiment 4.3 (lower). The lower left histograms show the cumulative recognition index (D1) scores (Trials 1–4) while the lower right histograms depict the total exploration times (Trial 0 only). In the high interference condition, the rats had previously been kept in the light with other objects. In the low interference condition, the rats had previously been kept in the dark. On Trial 0, rats were given an identical pair of novel objects to explore. Data shown are mean ± standard error. * *p* < .05.

In this experiment, rats performed an object recognition task when there was either a pair of intervening objects between the sample and test trial or there was no intervening object (none; Figure 4). The D1 data from Trials 1–4 were combined across the 2 sessions, giving a total of 4 trials with an intervening object and 4 none trials. Overall, for Trials 1–4, the rats with perirhinal lesions had lower D1 scores than the sham controls, *F*(1, 22) = 7.33, *p* = .013 (Figure 8, upper left). There was also a significant group by trial type interaction, *F*(1, 22) = 4.70, *p* = .041. The simple effects showed that the perirhinal lesion rats had lower D1 scores on the intervening object condition, *F*(1, 44) = 12.0, *p* = .001, but not on the no object (none) condition, *F* < 1, consistent with a greater disruption caused by the intervening object. Total exploration levels did not differ between the groups, *F*(1, 22) = 2.46, *p* = .13, or between the two interference conditions, *F* < 1 (Figure 8, upper right). [Fig-anchor fig8]

The D1 data from just Trial 1 were also examined, as any differential interference effects due to preceding objects should be most pronounced at the start of the session, although the analysis is weakened by the reliance on just 1 trial per condition. The pattern of results was similar to that seen when Trials 1–4 were grouped, although the effects were less clear-cut. There was evidence of a group by trial type interaction, *F*(1, 22) = 3.92, *p* = .061. In view of the research question and the results from Trials 1–4, simple effects were examined, despite the interaction not reaching significance. This analysis indicated that the D1 scores of the two groups did not differ for the no intervening object (none) condition, *F*(1, 44) = 1.14, *p* = .29, but the perirhinal rats were significantly worse for the intervening object condition, *F*(1, 44) = 4.30, *p* = .044.

#### Experiment 4.3 pretest manipulations of interference levels (waiting in the light or in the dark)

The different pretesting experiences in this experiment (in the light with two objects, high interference, or in the dark with no objects, low interference) affected subsequent behavior but did not appear to affect differentially the D1 scores of the rats with perirhinal lesions (Figure 8, lower). The rats explored the matching pair of novel objects in Trial 0 for longer when they had previously been housed in the dark than in the light, *F*(1, 22) = 22.79, *p* < .001 (Figure 8, lower right). There was, however, no effect of lesion, *F* < 1, and no interaction, *F*(1, 22) = 2.56, *p* = .12, on this measure as both the sham rats and rats with perirhinal cortex lesions showed greater exploration following prior experience in the dark (simple effects, both *p* < .05). On Trial 1, prior experience in the light appeared to reduce D1 scores, but this effect was not significant, *F*(1, 22) = 3.52, *p* = .074, nor was there an overall effect of lesion on the D1 scores for Trial 1, *F* < 1, or interaction between surgery and trial type, *F* < 1.

The same analyses were extended to the cumulative data from Trials 1–4 for the two pretesting conditions, although for these trials all rats were tested in the light and so increasingly experienced the same interference conditions. The D1 scores (Figure 8, lower left) did not distinguish the two groups, *F* < 1, nor was there an effect of condition, *F* < 1, or a group by interference interaction, *F*(1, 22) = 1.70, *p* = .21.

## Experiment 5. Exploration of Pairs of Novel Objects (Cohort 5)

In the final study, the rats only had pairs of novel objects to explore. The goal was to measure total exploration levels when the time spent with novel and the time spent with familiar objects were not in competition, given that the two behaviors cannot occur at the same time. It was anticipated that novel objects would create high levels of exploration.

### Materials and Method

#### Housing and surgery

Cohort 5 consisted of 31 rats with a weight range between 290 g and 340 g at the time of surgery. The housing conditions matched those of Cohort 3. Eighteen rats in Cohort 5 received bilateral perirhinal cortex lesions, while 13 rats were used as controls. The surgery was carried out in a very similar way to Cohort 2. The differences were that the volume of NMDA injected was 0.22 μl for the rostral injection and 0.20 μl for the middle and caudal injections. The injection coordinates (in mm) relative to bregma were also slightly different: (a) AP −1.8, ML ± 5.9, DV −9.3; (b) AP −3.4, ML ± 6.2, DV −9.5; (c) AP −5.0, ML ± 6.3, DV −8.9. The surgical control group received identical treatment, except that the dura was repeatedly perforated with the same Hamilton syringe, but no fluid was infused into the brain.

#### Behavioral procedure

Initial postoperative training comprised two tests of long-term object recognition and recency memory in the bow-tie maze (not reported). There was an interval of 14 weeks before testing for Experiment 5 began.

The rats were tested in the bow-tie maze, using standard procedures described above for object recognition (Experiments 1–4), but with one critical difference. Rather than be allowed to explore one novel object and one familiar object on each of the 20 trials, both objects were novel (Figure 1, lower). Consequently, every object used in Experiment 5 was novel.

### Results

#### Histology

The perirhinal cortex lesions in Cohort 5 were similar to those in the previous cohorts (see Figure 2). Consequently, there was almost complete cell loss along the length of the perirhinal cortex. This cell loss typically reached the junctions with the insula (rostral) and the postrhinal cortex (caudal). Eight of the 18 cases were excluded, however, as they had appreciable bilateral damage in the hippocampus (CA1 field) in addition to the targeted parahippocampal lesion. In the 10 remaining cases, the overall amount of tissue loss in the perirhinal cortex ranged from 67% to 98%. Of these cases, 5 had only unilateral cell loss in the CA1 field adjacent to the caudal part of the rhinal sulcus, while the remainder had extremely restricted CA1 cell loss (typically on just 1 or 2 sections) in the other hemisphere. In these same 10 cases, there was consistent cell loss in those parts of the piriform and lateral entorhinal cortices adjacent to the perirhinal cortex. In 4 of these 10 cases, there was unilateral damage in the most superior part of the lateral amygdala nucleus, while that part of Te2 closest to area 36 was also often partly damaged. One sham rat was excluded as it had unilateral damage to the rostral half of the dorsolateral cortex for unknown reasons. The final group numbers were therefore PRh5 = 10, Sham5 = 12.

#### Behavior

**Figure 9 fig9:**
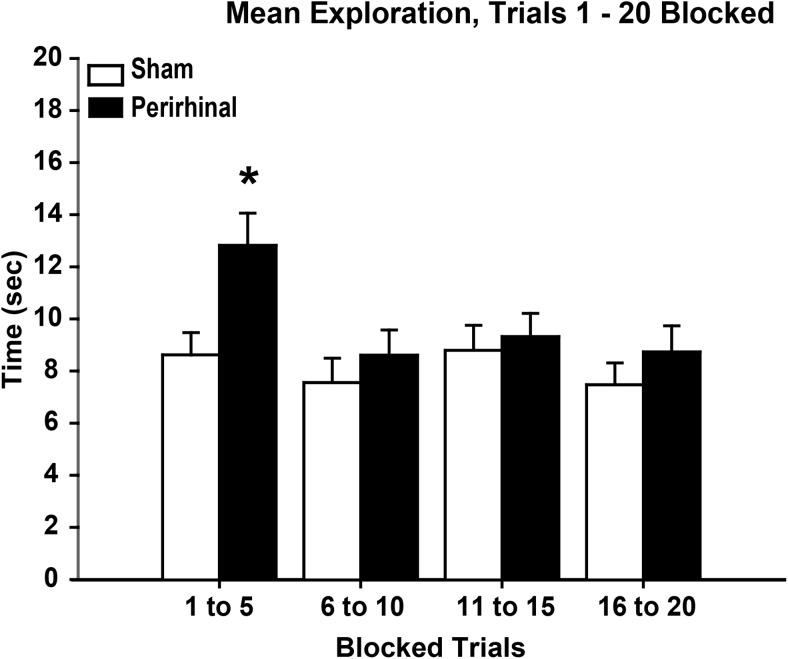
Experiment 5: Mean total exploration times when rats (Cohort 5) were given two different novel objects to explore on every trial. The data, which are blocked into four sets of 5 consecutive trials, show the mean amount of exploration (± standard error) on each trial. * *p* < .05.

This experiment compared levels of exploration for novel objects (see Figure 1, lower). When the data were separated into four blocks, each of 5 consecutive trials (see Figure 9), it became apparent that the levels of exploration for the two groups were highly comparable for blocks 2–4 (see Figure 9). The perirhinal lesion group did, however, show signs of heighted exploration across the first block of 5 trials (see Figure 9). A mixed analysis of variance (ANOVA) found no difference in the overall amount of object exploration made by the two groups, *F*(1, 20) = 2.28, *p* = .15. There was, however, an effect of trial order as the first block of 5 trials received the most exploration (overall effect of trial block l, *F*(3, 60) = 6.84, *p* = .001), as well as group by trial block interaction, *F*(3, 60) = 3.30, *p* = .026. This interaction reflected the raised levels of exploration shown by the rats with perirhinal lesions on the first block of trials, simple effects, *F*(1, 80) = 9.67, *p* = .003, while for the remaining three blocks there was no difference between the rats with perirhinal cortex lesions and their controls, all *F* < 1 (see Figure 9).[Fig-anchor fig9]

## Discussion

The present study quantified the impact of perirhinal cortex lesions on the exploration of novel and familiar objects. The first goal was to examine the prediction that rats with perirhinal lesions would show *false memories,* that is, treat novel stimuli as if they are familiar (McTighe et al., 2010; Romberg et al., 2012), so displaying unusually low exploration levels for novel objects. The second goal was to test the prediction that rats with perirhinal lesions are unusually sensitive to interference effects from objects in preceding trials, leading to a progressive failure of recognition and an enhanced reduction in exploration as test stimuli appear increasingly familiar (Cowell et al., 2010; McTighe et al., 2010; Romberg et al., 2012). For these reasons, exploration levels and recognition performance were compared along the course of a single session with multiple trials, during which proactive interference effects should increase (Experiments 1–4). Additional tests sought more specifically to manipulate levels of proactive interference (Experiments 4.2 and 4.3). The present study found only qualified evidence for the presence of false memories. Although there was support for the idea that perirhinal lesions can increase sensitivity to proactive interference, this occurred without the predicted exaggerated decline in exploration levels as trials (and proactive interference) accrue across a session.

In each of Experiments 1–4, the perirhinal cortex lesions impaired object recognition performance when measured across the entire session. This deficit occurred despite only testing short retention delays, which can reduce the impact of perirhinal lesions on recognition memory (Norman & Eacott, 2004; but see Bartko et al., 2007a). At the same time, each perirhinal lesion cohort performed above chance, though at much lower levels than the sham rats, that is, they could still distinguish the novel objects at an attenuated level. While the sham control rats sometimes showed greater overall object exploration (novel plus familiar) than the perirhinal lesion rats, this difference was only significant for Experiment 4, that is, in Experiments 1–3 there was no group difference in overall exploration. A much more consistent difference was that the sham rats spent more time exploring the novel objects than the perirhinal rats (Experiments 1–4). In Experiment 3, the complementary pattern was significant, here the PRh3 rats explored the familiar objects more than the Sham3 rats.

Despite some differences in prior training, the object recognition tests in Experiments 1–4 were conducted in the same way for all cohorts of rats. All 4 experiments represented the first recognition memory test postsurgery, ensuring that the rats had received limited prior object experience. Such similarities made it possible to analyze jointly all four groups, giving totals of 44 rats with perirhinal cortex lesions and 40 sham controls. The rats with perirhinal lesions were markedly impaired from the very first trial of the session, when proactive interference should be least. This initial deficit meant, however, that it could not be determined whether the relative scale of the recognition impairment, compared with the control rats, changed as the session progressed. Nevertheless, grouping the recognition index data by blocks of 5 consecutive trials revealed profiles of D1 scores for the perirhinal rats that closely shadowed those of the control rats, albeit at a lower level, without showing a growing disparity as the session progressed (Figure 7, upper). Floor effects occurred, however, for the final block of trials (Trials 16–20).

When data from Experiments 1–4 were combined and blocked (e.g., first 10 trials all 4 cohorts, 20 trials for 3 cohorts), it emerged that perirhinal cortex lesions can cause a small, but significant, reduction in overall object exploration. This small, overall reduction reflected the slightly greater increase in the exploration of novel stimuli by control rats compared with the relative increase in the exploration of familiar stimuli shown by rats with perirhinal lesions. Even so, in Experiment 5, when rats only explored novel objects, there was no evidence of a reduction in object exploration by rats with perirhinal lesions. In addition, there was no evidence that the rats with perirhinal lesions showed a progressive decrease in overall object exploration as the session continued that was greater than that seen in the control group (Experiments 1–5). This null result appears inconsistent with the prediction that as rats with perirhinal lesions encounter more and more objects, they should show an enhanced susceptibility to treat all objects as familiar, so reducing exploration (McTighe et al., 2010). This prediction stems from the assumption that the perirhinal cortex aids in the discrimination of stimuli with overlapping features (Bartko et al., 2007a; Bussey & Saksida, 2002; Bussey & Saksida, 2007; Bussey, Saksida, & Murray, 2002), a function that will be increasingly taxed as more and more stimuli are presented. While the present study did not deliberately introduce overlapping features, it is inevitably that the test objects shared some common elements. For this reason, the last pair of trials (19 + 20) is of particular interest. While the rats with perirhinal lesions showed a recognition deficit (D1), their levels of overall (novel plus familiar) exploration did not differ from the controls. Furthermore, the perirhinal cortex rats showed a significant drop in exploration of the novel objects that was countered by a significant increase in exploration of the familiar objects. Likewise, for Trials 6–10 (all 4 cohorts), the rats with perirhinal lesions showed significantly more familiar object exploration than the control rats. The latter behaviors appear inconsistent with the false memory description.

Despite finding that the rats with perirhinal lesions often showed only marginal reductions in overall exploration levels (Experiments 1, 2, 3) and no reduction in Experiment 5, there was evidence that recognition performance after perirhinal lesions can be particularly sensitive to increased interference. Exposing rats to intervening objects between a continuous series of recognition tests was especially disruptive to rats with perirhinal cortex lesions (Experiment 4.2). Furthermore, keeping rats in the dark before recognition testing (Experiment 4.3) not only increased object exploration for both perirhinal and sham rats, but also appeared to differentially benefit the rats with perirhinal lesions on the recognition trial. These results have clear parallels with those of McTighe et al. (2010), where holding rats in the dark between the sample and test phases restored the recognition performances of rats with perirhinal cortex lesions.

Given that perirhinal lesions disrupt object recognition, it could be assumed that the surgery either causes novel objects to appear familiar or familiar objects to appear novel. If all objects are perceived as novel (Wiig & Bilkey, 1994), there should be an increase in total exploration after perirhinal lesions. This pattern was not seen in Experiments 1–4. Although there was some evidence of increased initial object exploration after perirhinal cortex damage in Experiment 5, all of the objects in that study were novel. Instead, the dominant effect in the present series of studies was a marginal decrease in total exploration following perirhinal lesions. At first sight, this decrease appears consistent with a bias for rats with perirhinal lesions to perceive novel objects as familiar, that is, to show false memories (McTighe et al., 2010; Squire, Wixted, & Clark, 2007). Indeed, for Trial 1 of Experiments 1–4, the rats with perirhinal lesions showed reduced exploration of the novel object along with levels of familiar object exploration comparable with the controls, that is, the predicted pattern. However, these same rats did not show a decrease in overall exploration, while an increase in the exploration of familiar objects relative to the sham controls was seen across later trials, results not predicted by the false memory interpretation (McTighe et al., 2010).

These findings raise the important question of whether the results in Experiments 1–4 arose because each trial contained both a novel and a familiar object, that is, the two objects were in competition and so could not be explored at the same time. It is, however, difficult to see how this test arrangement would prevent the rats with perirhinal lesions from showing a marked reduction in overall exploration if stimuli rapidly appear familiar following immediately prior object experience (McTighe et al., 2010; Romberg et al., 2012). One possible explanation is that rats allocate a set amount of time for object exploration during each trial. As a consequence, the present pattern of results for perirhinal lesions (relative decrease for novel objects, relative increase for familiar objects so that the overall times are little changed) would still match the false memory predictions. This assumption does not, however, withstand scrutiny, as rats will readily vary the time spent exploring objects according to whether both objects are novel or both are familiar (Albasser et al., 2009, 2011a; McTighe et al., 2010). Furthermore, inspection of individual trials from the present study highlighted the variability in exploration times given to particular objects. A related point is that in Experiments 1–4, the total time spent exploring objects in a given trial comprised approximately 8 s of each 60-s trial, that is, a considerable amount of time within each trial was still potentially available for additional object exploration.

To resolve this issue, previous studies have given rats with perirhinal lesions pairs of either novel stimuli or pairs of familiar stimuli (Albasser et al., 2009, 2011a; McTighe et al., 2010; Mumby et al., 2007). In some cases, the rats explored pairs of the same novel object (Albasser et al., 2009; McTighe et al., 2010) while in other studies, the rats explored pairs of different novel objects (Albasser et al., 2011a). By only presenting novel or familiar stimuli, it is possible to remove any time competition between classes of objects. For the same reason, Experiment 5 examined exploration times for only novel stimuli. Compared with the sham controls, the rats with perirhinal lesions did not show reduced levels of exploration, nor did the lesioned rats show abnormally low levels of exploration as the 20 trials in the session progressed. These findings once again fail to support a false memory hypothesis that interacts with interference levels, though the behavioral design of Experimental 5 lacked a simultaneous contrast to demonstrate a concurrent, recognition memory deficit.

The results from previous studies that have provided pairs of novel stimuli to rats with perirhinal lesions have been inconsistent. As already noted, McTighe et al. (2010) found reduced exploration of novel objects in a Y maze on the test trial (but not sample trial). In contrast, seemingly normal levels of exploration to sets of novel objects were found when rats with perirhinal lesions were previously tested in the bow-tie maze (Albasser et al., 2011a: Experiment 1a, Trials 6–12; Experiment 1b, Trials 1–6.). This null result (Albasser et al., 2011a) is all the more striking as it occurred over multiple trials, when interference effects should increase. Likewise, Trial 0 of Experiment 4.3 looked at the exploration times for pairs of matching novel stimuli, but after previously being housed in the dark or housed in the light with an object. There was no evidence that the perirhinal lesions reduced novel object exploration after either condition when compared with the control rats.

Other relevant data come from the initial exploration of novel (sample) objects in an open field arena prior to subsequent object recognition performance. Normal levels of initial sample exploration by rats with perirhinal lesions have been repeatedly reported despite subsequent recognition deficits (Aggleton et al., 1997; Albasser et al., 2009; Barker et al., 2007, 2011; Ennaceur et al., 1996; Moran & Dalrymple-Alford, 2003; Mumby et al., 2007). This same pattern of normal sample exploration levels prior to recognition testing has also been reported for Y maze tasks (Bartko et al., 2007a,2007b; McTighe et al., 2010; Winters et al., 2004). The conclusion is, therefore, that rats with perirhinal lesions can show normal levels of exploration when first confronted with novel objects. To explain this pattern, it has been assumed that the home cage provides an impoverished environment offering little interference (Romberg et al., 2012). Consequently, the initial sample trial is interference free. As already noted, a number of experiments involving multiple trials within a session have found patterns of data inconsistent with this prediction (Albasser et al., 2011a, 2011b; current study, Experiment 5).

In conclusion, two predictions derived from representational-hierarchical models of perirhinal cortex function (Bussey & Saksida, 2002; Cowell et al., 2010) were examined. The first was that perirhinal lesions reduce exploration levels, consistent with treating novel objects as familiar, that is, the rats have false memories. The second was that perirhinal cortex lesions heighten sensitivity to visual interference. The present study gave only limited support for the first of these predictions as on many comparisons the perirhinal lesions increased the exploration of familiar objects. More support was found for the second prediction, with the qualification that increasing trial number within a session did not appear to disproportionately affect the exploration levels of rats with perirhinal lesions, even though proactive interference would increase.

The current results do, however, contain an apparent paradox. Rats with perirhinal lesions showed impaired object recognition yet still displayed exploration levels appropriate for novel stimuli when the novel objects were not placed in direct competition with familiar objects (Experiment 5). The implication is that there are extraperirhinal mechanisms that can signal the appropriate exploration levels for novel stimuli but which fail to assist the rat when directly choosing between a novel and a familiar object, that is, the perirhinal novelty/familiarity signal is tied to specific stimuli (Brown & Aggleton, 2001; Zhu, Brown, & Aggleton, 1995; see also Albasser et al., 2011a). Consequently, after a perirhinal cortex lesion, any remaining novelty signal becomes divorced from the correct stimulus, so the animal cannot link that signal to the specific object and make an appropriate recognition choice. Such a discrimination deficit is consistent with theories relating pattern separation to the perirhinal cortex (Bartko et al., 2007a; Saksida, Bussey, Buckmaster, & Murray, 2006). A number of candidate areas could generate less specific novelty and familiarity signals (Albasser et al., 2011a; Ho et al., 2011). Such information would mean that rats with perirhinal cortex lesions could, for example, still show seemingly normal stimulus habituation (Albasser et al., 2009, 2011a; Mumby et al., 2007) as long as the increasingly familiar stimulus is not put in competition with a novel stimulus. This same explanation would also predict that following perirhinal cortex lesions there should be a decrease in exploration of novel objects, but an increase in exploration of familiar objects when the two are exposed together, as a nonspecific signal of novelty remains. This pattern of results was often found when the experiments in the present study were combined. The slight bias to decrease overall exploration (Experiments 1–4 combined) may reflect the nonspecificity of that novelty signal.
